# An in silico analysis identifies drugs potentially modulating the cytokine storm triggered by SARS-CoV-2 infection

**DOI:** 10.1038/s41598-022-05597-x

**Published:** 2022-01-31

**Authors:** Laura Sanchez-Burgos, Gonzalo Gómez-López, Fátima Al-Shahrour, Oscar Fernandez-Capetillo

**Affiliations:** 1 Genomic Instability Group, Spanish National Cancer Research Centre, 28029 Madrid, Spain; 2 Bioinformatics Unit, Spanish National Cancer Research Centre, 28029 Madrid, Spain; 3 Science for Life Laboratory, Division of Genome Biology, Department of Medical Biochemistry and Biophysics, Karolinska Institute, 171 21 Stockholm, Sweden

**Keywords:** Computational biology and bioinformatics, Data mining, Cytokines, SARS-CoV-2

## Abstract

The ongoing COVID-19 pandemic is one of the biggest health challenges of recent decades. Among the causes of mortality triggered by SARS-CoV-2 infection, the development of an inflammatory “cytokine storm” (CS) plays a determinant role. Here, we used transcriptomic data from the bronchoalveolar lavage fluid (BALF) of COVID-19 patients undergoing a CS to obtain gene-signatures associated to this pathology. Using these signatures, we interrogated the Connectivity Map (CMap) dataset that contains the effects of over 5000 small molecules on the transcriptome of human cell lines, and looked for molecules which effects on transcription mimic or oppose those of the CS. As expected, molecules that potentiate immune responses such as PKC activators are predicted to worsen the CS. In addition, we identified the negative regulation of female hormones among pathways potentially aggravating the CS, which helps to understand the gender-related differences in COVID-19 mortality. Regarding drugs potentially counteracting the CS, we identified glucocorticoids as a top hit, which validates our approach as this is the primary treatment for this pathology. Interestingly, our analysis also reveals a potential effect of MEK inhibitors in reverting the COVID-19 CS, which is supported by in vitro data that confirms the anti-inflammatory properties of these compounds.

## Introduction

Since the first patient was hospitalized in Wuhan on December 12th, 2019^1^, the ongoing COVID-19 pandemic has caused 108.2 million confirmed infections and 5.3 million deaths by Dec 16th, 2021 (http://covid19.who.int). The disease-causing pathogen is SARS-CoV-2, a novel positive sense single-stranded RNA coronavirus belonging to the lineage B of the betacoronavirus genus^2^. Infections by different coronavirus strains have been historically present in humans, although they most often only cause a mild cold (e.g. infections by HKU, 229 and hCOV-OC43 coronaviruses)^3^. However, several new coronavirus strains have emerged in the last 2 decades that cause an Acute Respiratory Distress Syndrome (ARDS) with severe health consequences including patients’ death. These include SARS-CoV, which between 2002 and 2003 led to 8000 cases with a mortality of 9.5% and MERS-CoV, which in 2012 caused 2500 confirmed infections with a mortality rate of 36%^4^. As of today, and while there is significant variability between countries and final data are still to be fully defined, the averaged worldwide mortality associated to SARS-CoV-2 infections is around 2% (https://ourworldindata.org/mortality-risk-covid).

Due to the lack of a curative treatment for COVID-19, overwhelming efforts are being dedicated to the development therapies including vaccines^5^, plasma transfusions from convalescent patients^6^ and antiviral treatments limiting viral replication^7^ or infection^8,9^. Nevertheless, and while respiratory failure associated to ARDS is the leading cause of mortality in COVID-19 patients, accumulating evidence shows that the lethality in a subgroup of the severe patients occurs due to the late onset of an inflammatory “cytokine storm” (CS)^10^. First defined in 1993 in the context of graft-versus-host disease^11^, the CS has been observed in a wide range of inflammatory diseases such as multiple sclerosis or pancreatitis, and in the context of viral infections including SARS-Co^12,13^. In COVID-19, the CS is caused by an overproduction of proinflammatory cytokines such as IL-6, IL-1β and TNFα by lung infiltrating alveolar macrophages, which triggers vascular hyperpermeability, multiorgan failure and ultimately cell death^14^. Potential treatments for the CS of COVID-19 patients included broad spectrum anti-inflammatory therapies such as corticosteroids or more targeted therapies like anti-IL-6 receptor antibodies (Tocilizumab)^15^ which have been proven to improve survival and other clinical outcomes^16,17^. Other therapies like Janus Kinase (JAK) or IL-1 inhibitors have also been proposed as treatments for the CS based on their potent effects in suppressing key inflammatory signaling pathways driven by IFNγ or NFκB, respectively, and are being evaluated in clinical trials^18^. Importantly, the use of anti-inflammatory therapies such as corticosteroids is only recommended in COVID-19 patients undergoing a CS and not in mild cases, as the use of anti-inflammatory therapies in earlier stages of the disease would limit the efficacy of the immune system in fighting the infection.

Databases containing transcriptional signatures of human cancer cell lines exposed to a specific perturbation (perturbagen) such as drugs or genetic manipulations have become an excellent platform for data mining in biomedical research^19–21^. For instance, the *Connectivity Map* (CMap) from the Broad Institute at MIT stores over 1.5 M signatures from a wide range of human cancer cell lines exposed to around 5000 drugs and 3000 genetic perturbations (overexpression or shRNA-mediated depletion) (https://www.broadinstitute.org/connectivity-map-cmap)^22,23^. These signature databases can be used, for instance, to identify the mechanism of action of a drug, by comparing the transcriptional signature induced by the compound to that induced by genetic perturbation of individual genes^23^. Another relevant use of CMap is to identify drugs that induce a transcriptional signature which negatively correlates with that associated to a given disease as a strategy for discovering potential new therapies^20^. In the context of the COVID-19 pandemic, CMap mining has been used to identify drugs capable of modifying the levels of ACE2^24^, the receptor that SARS-CoV-2 uses for viral entry^8^. Here, and based on recently available transcriptomic data from COVID-19 patients, we used CMap to identify drugs that could potentially alleviate or aggravate the severity of the CS associated to late stages of this disease.

## Results

### Biological pathways related to the hypercytokinemia found in COVID-19 patients

**Figure 1:**
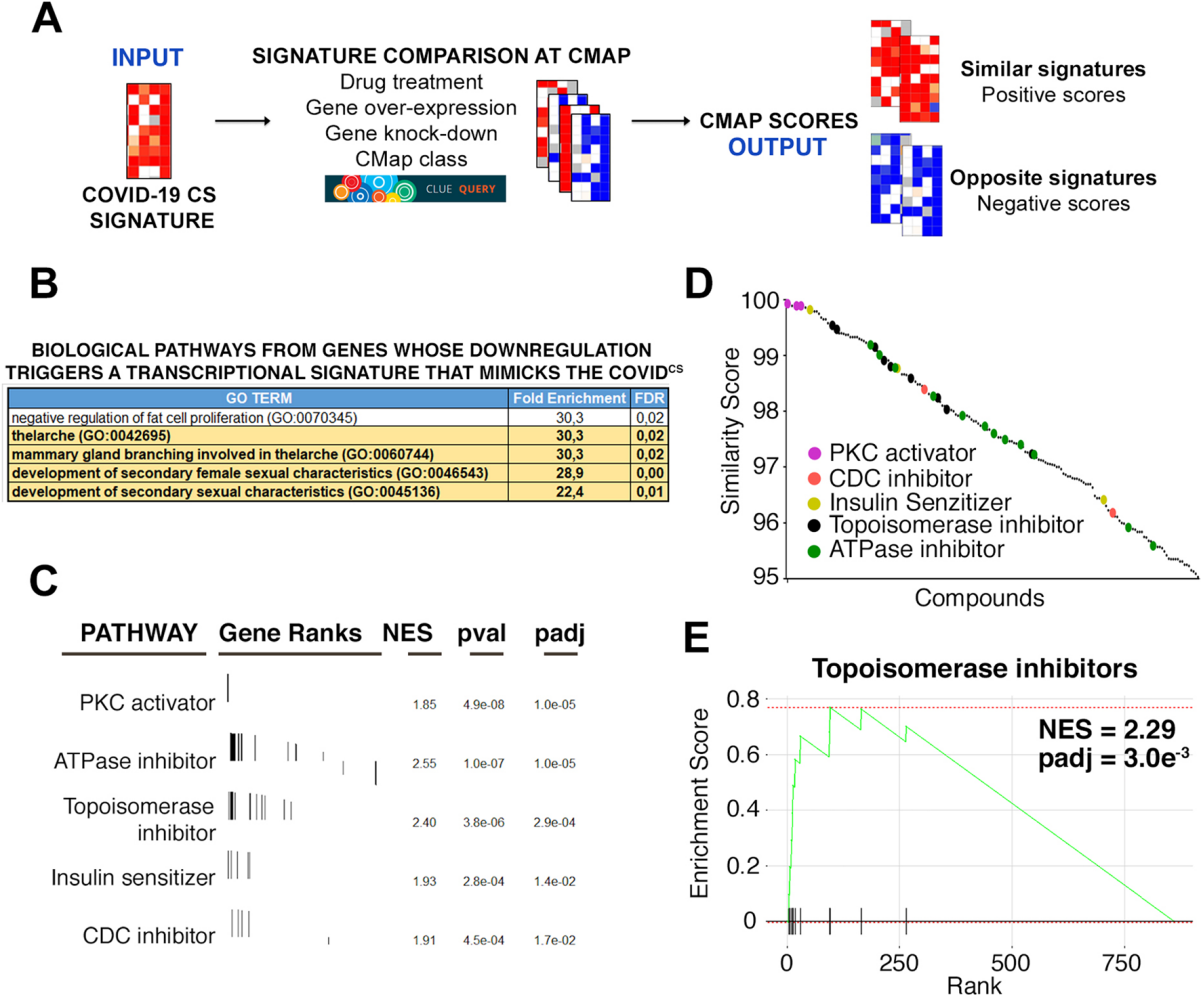
Biological pathways and compounds triggering transcriptional signatures that mimic the COVID^CS^. (**A**) Scheme of the analysis pipeline. Transcriptional signatures defined from COVID-19 CS data were queried into the clue.io tool from CMap (http://clue.io), to compare them to the signatures available at CMap linked to different perturbations (drug treatment, gene over-expression, gene knock-down and CMap class). The output of these analyses are similarity scores these being positive for signatures mimicking the COVID-19 CS signature, and negative for those opposing it. (**B**) Gene Ontology analysis of the biological pathways that are significantly enriched among genes for which their downregulation triggers transcriptional signatures that correlate to the COVID^CS^ (Similarity score > 95). The panel represents those with a False Discovery Rate (FDR) < 0.05 and a fold enrichment > 22.4. Pathways related to female hormone signaling are highlighted in yellow. (**C**) Drug GSEA analysis of compound classes triggering a transcriptional signature that positively correlates to the COVID^CS^. Compounds were classified by their mechanism of action. (**D**) Similarity scores of specific compounds with a COVID^CS^ similarity score bigger than 95. Compounds belonging to the classes defined in (**C**) are highlighted in different colors: PKC activators (purple), CDC inhibitors (pink), Insulin sensitizers (yellow), Topoisomerase inhibitors (black) and ATPase inhibitors (green). (**E**) Enrichment plot of Topoisomerase inhibitors resulting from the Drug GSEA analysis on medically approved compounds mimicking the COVID^CS^ signature.

In order to define a transcriptional signature related to COVID-19, we used recently published transcriptomic data from bronchoalveolar lavage fluid (BALF) cells where COVID-19 patients (n = 8) were compared to healthy individuals (n = 20) and community-acquired pneumonia patients (n = 146)^25^. Consistent with clinical observations, this study found that COVID-19 patients present a distinct activation of an IFN-dependent cytokine response. We thus defined a transcriptional signature based on the cytokines and cytokine receptor genes that were differentially expressed in COVID-19 patients (COVID^CS^) (signature available in Table S1). We then mined CMap using its clue.io tool (http://clue.io), in order to compare the COVID^CS^ with transcriptional signatures associated to specific perturbations (drug treatments, gene overexpression or knockdowns and biological pathways (CMap classes)). The output of these analyses are similarity scores, which are positive for those signatures that are similar to COVID^CS^ and negative for perturbations driving transcriptional signatures opposed to it (Fig. 1A).

We first looked into CMap classes with transcriptional signatures that correlate positively with the COVID^CS^. Consistent with their key roles in inflammation^26^, these analyses identified activation of NFκB and PKC signaling as the biological pathways with associated signatures that are most significantly similar to the COVID^CS^ (Table S2). Of note, PKC activation triggers NFκB and TNFα-dependent inflammatory responses in human bronchial epithelial cells, suggesting that PKC could act early on in triggering the inflammatory response^27^. Regarding specific genes our analysis identified IL1 (IL1R1) or TNFα receptors (TNFRS10A and TNFRSF1A) and IFN-response genes (IRF2 and IFR5) as the factors whose overexpression triggers a transcriptional signature that most significantly mimics the COVID^CS^ (Table S3). Thus, as expected, the transcriptional signature of the hypercytokinemia triggered by SARS-CoV-2 infection resembles that of activated inflammatory pathways regulated by PKC, NFκB, TNFα and IL1.

In what regards to genes that, when downregulated, trigger a signature similar to the COVID^CS^, the list was not as restricted to immune factors as the one from overexpressed genes, although it included anti-inflammatory factors such as NFκB inhibitor alpha (NFKBIA) (Table S4). Interestingly, Gene Ontology (GO) analyses of genes whose downregulation triggers a transcriptional signature mimicking that of the COVID^CS^ detected an enrichment in pathways related to female sexual hormones (Fig. 1B). We find this of particular interest since even if male and females are equally infected with SARS-CoV-2 the fatality rate is significantly higher in men, which could relate to the well-established gender-related differences in the intensity of inflammatory responses^28^. In fact, several studies have indicated that estrogen levels influence the severity of diseases linked to inflammation, including cancer, due the modulation of NFκB signaling^29–32^. Moreover, gender differences in COVID-19 mortality rates are biggest in younger patients and decline progressively, further supporting a connection with female hormone levels. Noteworthy, estrogen treatments are already been explored in clinical trials for reducing the severity of COVID-19 in men and women 55 and older (as estrogen levels decline after menopause)^33^.

### Drugs with transcriptional signatures that mimic the COVID^CS^

Next, we interrogated CMap for the identification of compounds triggering a similar effect on the transcriptome to the one observed in COVID-19 patients, as these compounds could in principle potentiate the severity of the hypercytokinemia. Consistent with the analysis of biological patwhays, three of the top compounds were PKC activators: prostratin, phorbol-12-myristate-13-acetate (PMA) and ingenol (Table S5). Besides specific compounds, we aimed to identify compound classes with signatures mimicking the COVID^CS^, for which we adapted Gene Set Enrichment Analyses (GSEA) to conduct a “drug GSEA”^24,34^. PKC activating drugs were the most significantly enriched class, followed by “ATPase inhibitors”, “Topoisomerase Inhibitors”, “Insulin sensitizers” and “CDC inhibitors” (Fig. 1D,E). Regarding insulin, a recent study has found that the triglyceride and glucose index (TyG) correlates with the severity and mortality of COVID-19 patients, which builds upon epidemiological observations identifying obesity as a risk factor among COVID-19-related fatalities^35^. If we restrict our analysis to medically approved drugs, only Topoisomerase inhibitors showed a significant enrichment as drugs mimicking the COVID^CS^ (Fig. 1E and Table S6), although the absence of other compound classes from this list can be influenced by the low number of clinically available drugs in other pathways. As for the potential aggravating effects of topoisomerase inhibitors on the COVID-19 CS, we believe that this is most likely related to Topoisomerase-II. In fact, Topoisomerase-I activity has been found to mediate the expression of inflammatory genes^36^. In contrast, Topoisomerase-II inhibition triggers the expression of inflammatory cytokines through activation of cGAS-STING innate immune sensing of cytoplasmic DNA^37^.

### Drugs triggering transcriptional signatures opposed to the COVID^CS^

**Figure 2:**
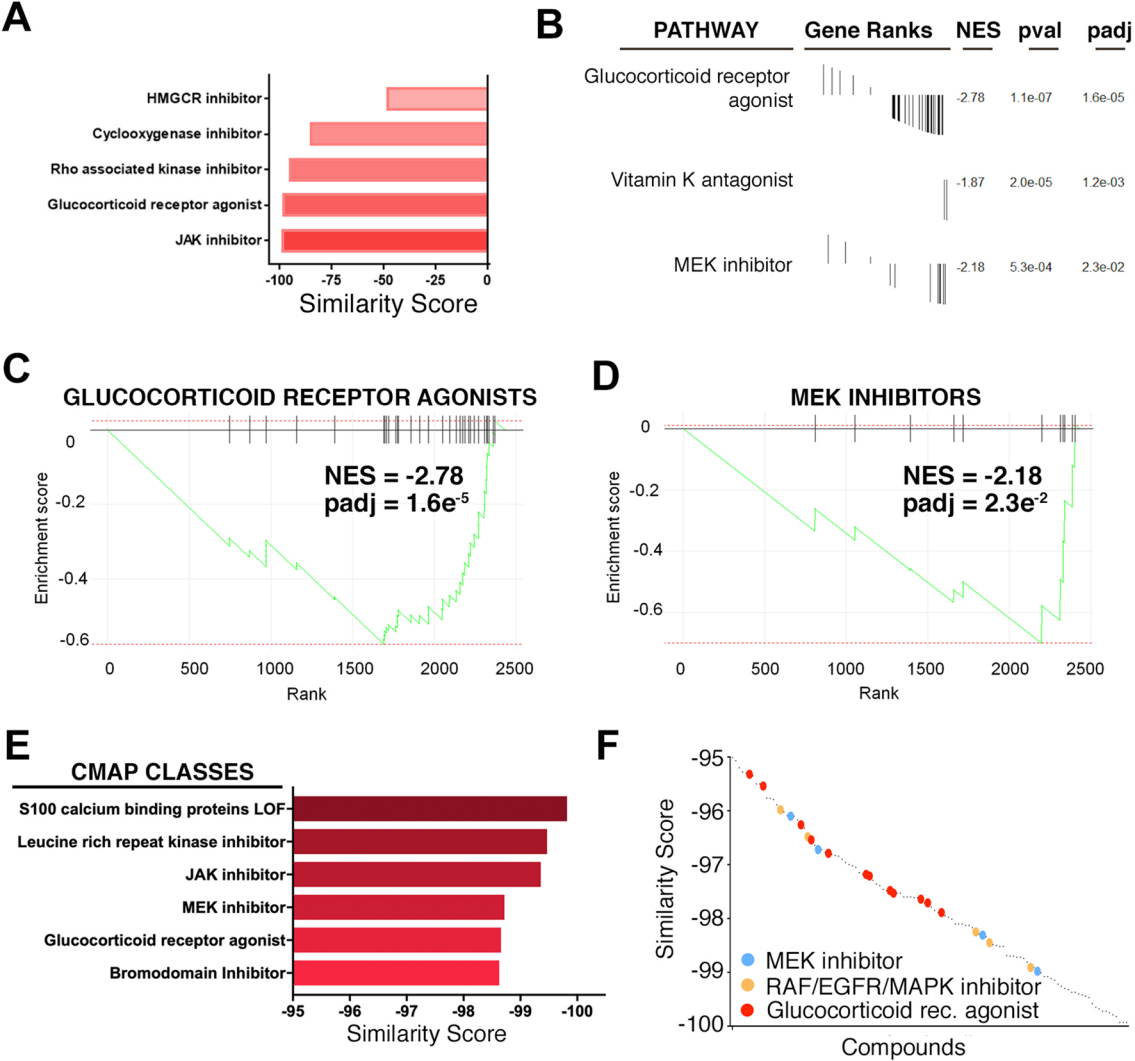
Compounds triggering transcriptional signatures opposed to the COVID^CS^. (**A**) COVID^CS^ similarity scores for drug types (CMap Classes) already under study for the treatment of the COVID-19 CS. (**B**) Drug GSEA analysis of compound classes with a transcriptional signature that negatively correlates to the COVID^CS^. Enriched pathways with padj value < 0.05 are shown, as well as their gene ranks, Normalized Enrichment Scores (NES), p-value (pval) and adjusted p-value (padj). (**C**, **D**) Enrichment plots of Glucocorticoid receptor agonists (**C**) and MEK inhibitors (**D**) resulting from the Drug GSEA analysis mentioned in (**B**) illustrating the overall negative correlation between the transcriptional signatures triggered by these compounds and the COVID^CS^. (**E**) Similarity scores of the top CMap Classes with an associated transcriptional signature that negatively correlates to the COVID^CS^. (**F**) Similarity scores of specific compounds with a COVID^CS^ similarity score lower than − 95. MEK inhibitors (blue), RAF/EGFR/MAPK inhibitors (orange) and glucocorticoid receptor agonists (red) are highlighted by the indicated colors.

As mentioned in the introduction, one of the initial ideas behind the development of CMap was to identify drugs that trigger transcriptional responses that negatively correlate with those associated to a given disease, in order to identify potential new therapies through drug repurposing^20^. Based on this concept, we next interrogated CMap for drugs that trigger signatures opposed to the COVID^CS^. Remarkably, all compound classes that have been already proposed for the treatment of the COVID-19 CS including corticosteroids and JAK or HMGCR inhibitors had transcriptional signatures that negatively correlated with the CS^s^^13,15,18,38,39^ (Fig. 2A and Table S7). In fact, drug GSEA analyses identified glucocorticoid receptor agonists as the compounds that trigger a signature most significantly opposed to the COVID^CS^, providing strong support to the usefulness of our approach as the synthetic glucocorticoid dexamethasone is currently the most widely used treatment for the CS in COVID-19 patients (Fig. 2B,C). Besides glucocorticoids, this analysis identified Vitamin K antagonists and MEK inhibitors as potentially counteracting the COVID^CS^, although the analysis of Vitamin K antagonists was based on very few compounds (Fig. 2B,D). An independent analysis of CMap classes confirmed glucocorticoid receptor agonists and MEK inhibitors as negatively correlating to the COVID^CS^ as well as other drugs in clinical trials for the treatment of the COVID-19 CS like JAK inhibitors^15,18^ (Fig. 2E and Table S7).

Other significantly enriched CMap classes include loss-of-function of S100 calcium-binding proteins and inhibitors of Bromodomains or Leucine-rich repeat kinases (LRRK), and existing literature supports their potential in counteracting a CS. First, S100 calcium-binding proteins and LRRK2 are known to promote inflammation and cytokine production^40–44^. In fact, an increase of S100 calcium-binding proteins has been reported in severe or critically ill COVID-19 patients^45,46^. Furthermore, treatment with paquinimod, a selective inhibitor of S100A8/A9, prevented death in a lethal model of mouse coronavirus infections^47^. As for bromodomain inhibitors, these have been proven to reduce inflammation and cytokine production in multiple models^48–51^. Remarkably, bromodomain inhibitors were recently shown to prevent inflammation-induced cardiac dysfunction and death in a mouse cytokine-storm model of SARS-CoV-2 infection^52^. Altogether, these evidences support that the validity our approach to identify inhibitors that could potentially reduce the severity of COVID^CS^.

Among these different types of compounds, the potential effects of MEK inhibitors in counteracting the cytokine response seem of particular interest for several of reasons. First, because even if MEK inhibitors have been mainly developed as antineoplastic agents, numerous studies support that targeting MEK limits cytokine responses in various contexts like graft-vs-host disease, cerebral ischemia, activation of T cells by superantigens or influenza A infection^53–58^. Second, because MEK inhibition has been previously shown to inhibit replication of other coronaviruses such as the mouse hepatitis virus (MHV)^59^. Third, because besides MEK, several compounds with the highest scores of opposing signatures to the COVID^CS^ are inhibitors of other factors from the same signaling route as MEK (RAF, EGFR and MAPK inhibitors) (Fig. 2F and Table S8). Finally, and most interestingly, there is actually an ongoing clinical trial exploring the efficacy of a MEK inhibitor (ATR-002) for the treatment of COVID-19 and its associated CS^60^.

### Validation of CMap analyses in two additional COVID-19 signatures

In order to complement our analyses, we defined another two transcriptional signatures associated to the COVID CS. First, we defined a transcriptional signature specifically related to severe patients undergoing a CS, for which we used data from a manuscript where the BALF transcriptome of a COVID-19 patient suffering from a CS was compared to that of healthy individuals or a milder case of the disease (signature available in Table S1; CS^s^)^61^. Second, we used a study where Liao et al*.* reported single-cell RNA-seq data from BALF samples obtained from healthy individuals and from COVID-19 patients with either mild disease or CS symptoms^62^. Using transcriptomic data from the lung-infiltrating macrophages analyzed in this study (6 patients), we defined a new transcriptional signature associated to the COVID-19 CS (signature available in Table S1; CS^s^[sc]). Consistent with our previous analyses, NFκB and PKC signaling were the CMap classes most significantly similar to the CS^s^ and to CS^s^[sc] (Fig. S1A,B and Table S2). Furthermore, TNFα receptors and IFN-response genes were once again found among the top list of factors that, when overexpressed, trigger a transcriptional signature similar to the CS^s^ (Table S3). As for genes that, when downregulated, trigger a transcriptional signature similar to the CS^s^ and to CS^s^[sc], this list also showed a significant overlap with that resulting from the COVID^CS^ analyses (Table S4). Importantly, GO analyses found once again an enrichment of pathways related to female hormone signaling among those that, when downregulated, trigger transcriptional signatures signatures that mimick the CS^s^ (Supp. Fig. S1C), further reinforcing the idea that the anti-inflammatory properties of estrogens might be behind the gender differences found on the severity of COVID-19. Despite the disparity of the datasets used, there was a significant overlap between the specific compounds eliciting a transcriptional signature that positively correlates with both the CS^s^, CS^s^[sc] and COVID^CS^ (Fig. S1D and Table S5).

We next queried CMap for drugs causing opposing signatures to the CS^s^ and to CS^s^[sc]. Once again, there was a significant overlap between the top compounds identified in these analyses with those from the COVID^CS^ (Fig. S2A). Still, the analysis yielded interesting differences with the previous COVID^CS^ analysis when we looked into specific compounds with signatures opposite to the CS^s^ (Table S8). In addition, the CS^s^[sc] the top hits included compounds not identified in whole transcriptome analyses and that could potentially be related to counteracting inflammation in specific cell types, including the estrogen estriol, relevant in the context of the above-mentioned gender-related differences in COVID-19 mortality. Nevertheless, drug GSEA analyses once again identified glucocorticoid receptor agonists as triggering transcriptional signatures that had a significant negative correlation with the CS^s^, together with inhibitors of Bromodomains, V Type ATPases and Leucine-rich repeat (LRRK), JAK or MEK kinase inhibitors (Fig. S2B and Table S7). Drug GSEA analysis also revealed MEK inhibitors together with “Bacterial cell wall synthesis inhibitors” and PI3K inhibitors as the most significantly enriched classes of compounds with a negatively correlating transcriptional signature to the CS^s^[sc] (Fig. S2C). Likewise, the analysis of CMap classes negatively correlating to the CS^s^[sc] had MEK inhibitors at the top of the list, together with loss of function of aminoacyl-tRNA synthetases and inhibitors of the reverse transcriptase, bacterial 30S ribosome, PKA and glycogen synthase kinase (Fig. S2D and Table S7).

### Anti-inflammatory properties of MEK and ERK inhibitors

**Figure 3:**
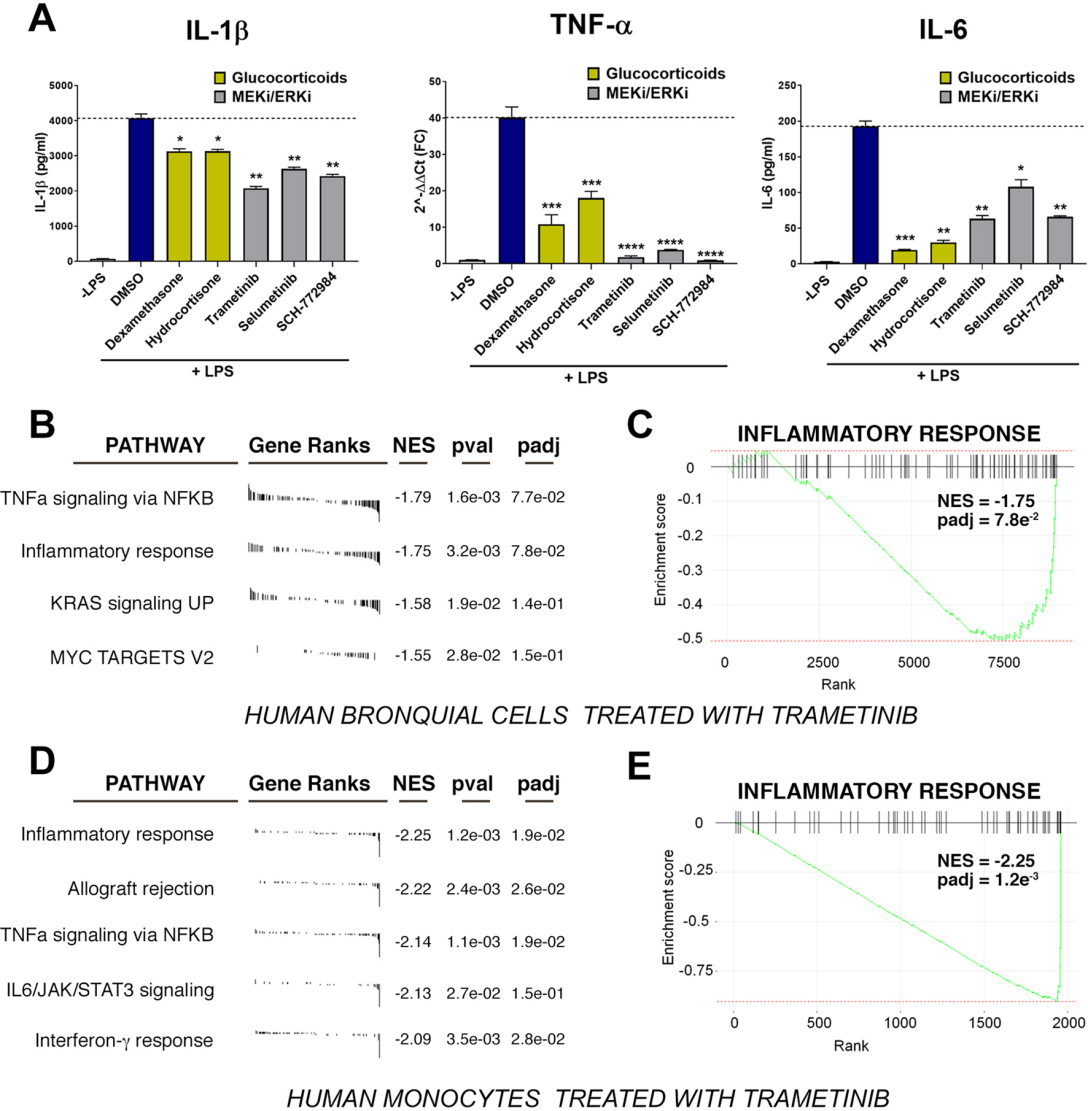
Inhibition of cytokine production by MEK and ERK inhibitors. (**A**) Cytokine expression in macrophages differentiated from THP1 cells upon exposure to LPS, with or without glucocorticoids (dexamethasone and hydrocortisone), MEK inhibitors (trametinib and selumetinib) and an ERK inhibitor (SCH-772984). IL-6 and IL-1β levels were measured by ELISA (pg/ml), and TNF-α by qPCR (2^−ΔΔCt^ (FC)). The experiment was repeated three times and a representative example is shown. *p < 0.05, **p < 0.01, ***p < 0.001, ****p < 0.0001 (*t* test). (**B**) GSEA analysis from human bronquial cells treated with trametinib or DMSO as a control. Differential expression analyses were done from data publicly available at GEO (GSE63229). Pathways names together with their gene ranks, Normalized Enrichment Scores (NES), p-values (pval) and adjusted p-values (padj) are shown. (**C**) Enrichment plot of genes belonging to the “Inflammatory response” pathway resulting from the GSEA analysis mentioned in (**B**) illustrating the inhibition of their expression levels by trametinib in human bronchial cells. (**D**) GSEA analysis from human monocytes treated with trametinib or DMSO as a control. Differential expression analyses were done from data publicly available at GEO (GSE123574). Pathways names together with their gene ranks, Normalized Enrichment Scores (NES), p-values (pval) and adjusted p-values (padj) are shown. (**E**) Enrichment plot of genes belonging to the “Inflammatory response” pathway resulting from the GSEA analysis mentioned in (**D**) illustrating the inhibition of their expression levels by trametinib in human monocytes.

As the efficacy of glucocorticoid receptor agonists for the treatment of the COVID-19 CS is well established, we decided to complete our study by investigating the potential anti-inflammatory properties of inhibitors of the MAPK/MEK/RAF route, which as mentioned have been primarily developed as anticancer agents. First, in vitro, we differentiated cells from a human monocyte cell line (THP-1) into macrophages, and triggered cytokine production by exposing them to lipopolysaccharide (LPS), an endotoxin found in the outer membrane of Gram-negative bacteria. Differentiated THP-1 cells were exposed to LPS together with two independent inhibitors (trametinib and selumetinib), one ERK inhibitor (SCH-772984) and two glucocorticoids (dexamethasone and hydrocortisone). The treatment with LPS promoted the activation of an inflammatory program, exemplified by an increased expression of cytokines such as IL-1β, TNF-α and IL6 (Fig. 3A). As expected, cytokine expression was reduced upon treatment with glucocorticoids. Strikingly, MEK and ERK inhibitors not only did they reduce LPS-induced cytokine production, but they actually had a bigger effect than glucocorticoids on IL-1β and TNF-α (Fig. 3A). Of note, we also tested the effect of inhibitors of RAF (vemurafenib) or EGFR (gefitinib and erlotinib), which act upstream of MEK and ERK in growth factor signaling. However, these compounds only showed partial effects on IL-6 and did not affect the expression of IL-1β or TNF-α (Fig. S3).

Finally, we explored if the effects of MEK inhibition in counteracting cytokine expression are also observed in cell types that are involved in the inflammatory responses triggered by COVID-19 infection. To this end, we took advantage of publicly available GEO data from human bronquial cells (GSE63229) and human monocytes (GSE123574) treated with either DMSO or trametinib (MEK inhibitor) to perform differential expression followed by Gene Set Enrichment Analyses (GSEA). Consistent with our THP-1 data, GSEA from human bronquial cells revealed that the pathways most significantly downregulated in trametinib-treated cells were “TNF-α signaling via NFκB” and “inflammatory response”, together with the expected downregulation of Ras signaling (“KRAS signaling up”) and “MYC targets” (Fig. 3B,C). Likewise, GSEA in human monocytes showed these two pathways, plus “allograft rejection”, “IL6-JAK-STAT3 signaling” and “interferon-γ response” as the ones being most repressed by trametinib (Fig. 3D,E). Collectively, these experiments provide support to the concept of using inhibitors of MEK and ERK kinases to alleviate pathologies linked to hypercytokinemia such as the CS that evolves in severe cases of COVID-19.

## Discussion

We here present our results using large-scale public repositories of transcriptional signatures and bioinformatics tools to identify compounds and genetic perturbations that trigger a transcriptional response similar or opposite to that observed in severe patients of COVID-19 undergoing a CS. We are aware of the caveats of the limited data available to perform these studies at this point, which should get stronger as more transcriptomic data from these patients becomes available. We find nevertheless remarkable the large degree of overlap that we found in our analyses, despite using three different datasets and two different models (whole tissue vs single-cell) to define our transcriptional signatures. Besides identifying drugs that are already being used (e.g. glucocorticoid receptor antagonists) or are being tested in clinical trials (e.g. JAK inhibitors) as COVID-19 therapies, our study supports the potential for drug repositioning of compounds like MEK or ERK inhibitors for treating the COVID-19-associated CS, which in the case of MEK is already being tested in clinical trials. It is interesting to note that the effects of MEK inhibitors in suppressing hypercytokinemia seem independent from their well-established antiproliferative roles that relate to cancer therapy, as the anti-inflammatory effects we observed in THP-1 cells were made in differentiated non-growing cells. We must again emphasize that, in any case, any therapy based on anti-inflammatory drugs should be restricted to the late and severe stages of COVID-19, as their use could limit the efficacy of the immune system in fighting the infection at earlier stages.

Besides identifying compounds that could potentially be of use for treating severe cases of COVID-19, we raise awareness on the possible adverse effects that some drugs like Topoisomerase inhibitors might have in potentiating the CS. In this regard, our manuscript supports that the gender-related differences on the severity of COVID-19 might be related to the anti-inflammatory properties of female hormones. We want to end this manuscript with a clear statement in that by no means we are proposing novel clinical indications for any of these agents. We simply wanted to contribute in the context of the current health crisis and provide a resource that others might find of use in the selection of drugs that could be tested for efficacy in experimental models of CS.

## Materials and methods

### Calculation of similarity scores

Three independent transcriptional signatures defined from COVID-19 patients (COVID^CS^, CS^s^ and CS^s^[sc]) were used as inputs to query CMap using its clue.io tool (https://clue.io), in order to obtain similarity scores for the signatures associated to all the perturbagens (compounds, CMap classes and the over-expression or knock-down of genes) available at CMap^23^. The Query CMap tool was used, employing Gene expression (L1000), Touchstone and individual query as query parameters. Similarity scores were downloaded, and results sorted based on their scores and the type of perturbagen. For restricting the analysis to medically approved drugs, we used drug information available at CMap.

### Gene ontology (GO)

For GO analyses, a list of genes in which their perturbation (overexpression or knockdown) led to a similarity score > 95 with COVID-19-associated signatures was used as input in "The Gene Ontology Resource", release 2020-04-23 (http://geneontology.org/)^63–65^.

### Drug “Gene Set Enrichment Analysis” (GSEA)

We adapted Gene Set Enrichment Analysis (GSEA) to enable enrichment analyses of "drug classes" based in their mechanism of action (MOA), for which we used the GSEA method implemented in the R package fgsea^34^, similarly as done by Sinha et al.^24^. In brief, similarity scores from the signatures associated to all compounds were ranked and the GSEA method applied to the ranked list with “gene sets” for GSEA analysis, being “gene sets” the sets of drugs within each class. Drug classes were defined based on their mechanisms of action (MOA); for which annotation data was included in CMap analysis as “description”. R version 3.6.3 (2020-02-29) was used for computational analyses and a sample code can be obtained at https://github.com/Genomic-Instability-Lab/An-in-silico-analysis-of-drugs-potentially-modulating-the-cytokine-storm-triggered-by-SARS-CoV-2-inf.

### Gene Set Enrichment Analysis (GSEA) for GEO databases

GEO data from human monocytes (GSE123574) and bronquial human cells (GSE63229) treated with DMSO or the MEK inhibitor trametinib was used to perform Gene Set Enrichment Analyses (GSEA). Differential expression data was obtained from the original paper in the case of the human monocytes^66^. For bronquial cells differential expression analysis was performed using the GEO2R GEO interface. The GSEA method implemented in the R package fgsea^34^ was used. Specifically, the gene expression log fold change values were ranked, and the GSEA method was applied to the ranked list with the Hallmark Signatures obtained from the GSEA database (https://www.gsea-msigdb.org/gsea/index.jsp). R version 3.6.3 (2020-02-29) was used for computational analysis and a sample code can be obtained at https://github.com/Genomic-Instability-Lab/An-in-silico-analysis-of-drugs-potentially-modulating-the-cytokine-storm-triggered-by-SARS-CoV-2-inf.

### In vitro assessment of LPS-induced inflammatory signaling

THP-1 human monocyte cells were maintained in RPMI 1640 medium containing 10% FBS and 1% penicillin/streptomycin, and grown at 37 °C in a humidified air atmosphere with 5% CO_2_. Cells in logarithmic growth phase were seeded as 2 × 10^6^ cells in 10 cm dishes and differentiated into macrophages after incubation with 100 ng/mL PMA for 48 h. Differentiated cells were subsequently washed with PBS and grown in fresh RPMI 1640 medium for another 24 h. Cells were then pre-treated for 1 h with either DMSO (control) or 10 μM of each drug, followed by a 6 h treatment again with either DMSO or 10 μM drugs in the absence or presence of 500 ng/mL LPS. Cell supernatants and pellets were obtained for ELISA and q-RT-PCR analyses, respectively. The following compounds were purchased from Selleckchem: Phorbol 12-myristate 13-acetate (PMA) (S7791), trametinib (S2673), selumetinib (S1008), SCH-772984 (S7101), vemurafenib (S1267), gefitinib (S1025) and erlotinib (S7786). LPS (L2630), dexamethasone (D4902) and hydrocortisone (3867) were purchased from Sigma.

### ELISA

Cell culture supernatants were centrifuged at 10,000*g* for 1 min at 4 °C and analysed with IL-6 and IL-1β immunoassay kits (Invitrogen) according to the protocols provided by the manufacturer. LPS-treated supernatants had to be diluted prior analysing: 1:5 for IL-6 detection, and 1:50 for IL-1β. Non-LPS-treated samples were not diluted for IL-6 detection and a 1:10 dilution was used in the case of IL-1β immunoassay. The absorbance of each well at 450 nm was measured and analysed using a PerkinElmer VICTOR Nivo Multimode Plate Reader and the MyAssays Desktop Explorer Software respectively.

### Quantitative RT-PCR

Total RNA was extracted using the Agilent Absolutely RNA Miniprep Kit following manufacturer’s instructions. mRNA levels were measured by real-time quantitative PCR after reverse transcription of RNA, using for both the Invitrogen SuperScript III Platinum SYBR Green One-Step qRT-PCR Kit with ROX. Quantitative RT-PCR was performed on an Applied Biosystems QuantStudio 6 Flex Real-Time PCR System. We used the following primers sequences: GAPDH (GGACTCATGACCACAGTCCATGCC, TCAGGGATGACCTTGCCCACAG) and TNF-α (TTGTAGCAAACCCTCAAGCTGA, AGATGAGGTACAGGCCCTCTGA. The levels of GAPDH mRNA were used as control to normalize expression values.

### Graphical representation and statistical analysis

“Drug” GSEA and GSEA graphs were obtained with R as explained in their corresponding section. Venn diagrams were represented using BioVenn^67^, and the statistical significance for the overlap was calculated using the superexact test^68^. For the rest of the graph representations and the statistical analysis we used GraphPad Prism version 7.04.

## Supplementary Information

Supplementary Figures.

Supplementary Tables.
