# Supplementary material for: An in silico analysis identifies drugs potentially modulating the cytokine storm triggered by SARS-CoV-2 infection
Source: Sci Rep. 2022 Jan 31;12:1626. doi: 10.1038/s41598-022-05597-x (PMC8803893; doi:10.1038/s41598-022-05597-x)
Supplement: Supplementary file 1 — Supplementary Figures. [file 41598_2022_5597_MOESM1_ESM.pdf]

A

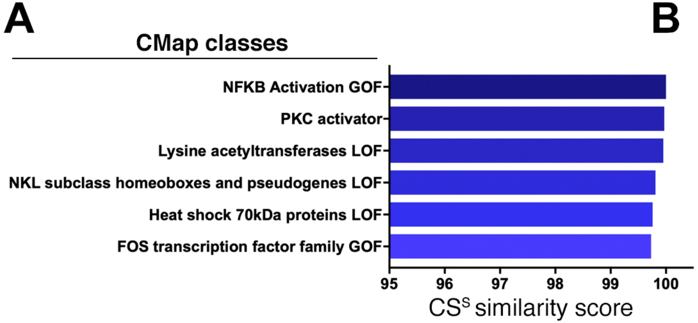

B

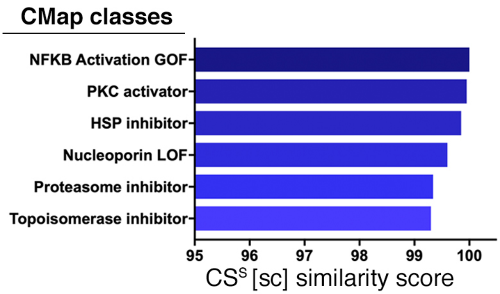

C

GO TERM	Fold Enrichment	FDR
abortive mitotic cell cycle (GO:0033277)	50,2	0,05
positive regulation of timing of anagen (GO:0051885)	50,2	0,05
Schwann cell proliferation (GO:0014010)	50,2	0,05
negative regulation of oxidative phosphorylation uncoupler activity (GO:2000276)	50,2	0,05
adenine salvage (GO:0006168)	50,2	0,05
nucleotide-binding oligomerization domain containing 1 signaling pathway (GO:0070427)	50,2	0,05
chemorepulsion of dopaminergic neuron axon (GO:0036518)	50,2	0,05
histone H3-T6 phosphorylation (GO:0035408)	50,2	0,05
cholesterol esterification (GO:0034435)	37,7	0,01
sterol esterification (GO:0034434)	37,7	0,01
steroid esterification (GO:0034433)	37,7	0,01
thelarche (GO:0042695)	30,2	0,01
mammary gland branching involved in thelarche (GO:0060744)	30,2	0,01
development of secondary female sexual characteristics (GO:0046543)	28,7	0,00
development of secondary sexual characteristics (GO:0045136)	27,9	0,00
mammary gland involution (GO:0060056)	25,1	0,02
chemorepulsion of axon (GO:0061643)	25,1	0,02

BIOLOGICAL PATHWAYS FROM GENES WHOSE DOWNREGULATION TRIGGERS A TRANSCRIPTIONAL SIGNATURE THAT MIMICKS THE CS^{S}

D

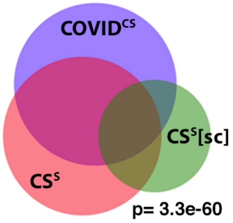

TOP COMPOUNDS WITH SIGNATURES MIMICKING COVID^{CS}, CS^{s} AND TO CS^{s}[sc]

Figure S1. Biological pathways and compounds triggering transcriptional signatures that mimic CS^{S} and CS^{S}[sc]. (**A**) Similarity scores of the top biological pathways (CMap Classes) with associated transcriptional signatures that show a positive correlation to the CS^{S}. (**B**) Similarity scores of the top CMap Classes with associated transcriptional signatures that show a positive correlation to the CS^{S}[sc]. (**C**) Gene Ontology analysis of the biological pathways that are significantly enriched among genes for which their downregulation triggers transcriptional signatures that positively correlate to the CS^{S} (Similarity score > 95). The panel represents those with a False Discovery Rate (FDR) <0.05 and a fold enrichment >25.1. Pathways related to female hormone signaling are highlighted in yellow. (**D**) Venn Diagram representing the overlap among the top compounds that trigger transcriptional signatures that positively correlate to COVID^{CS}, CS^{S} and CS^{S}[sc]. The p-value was obtained with the *superexact* test.

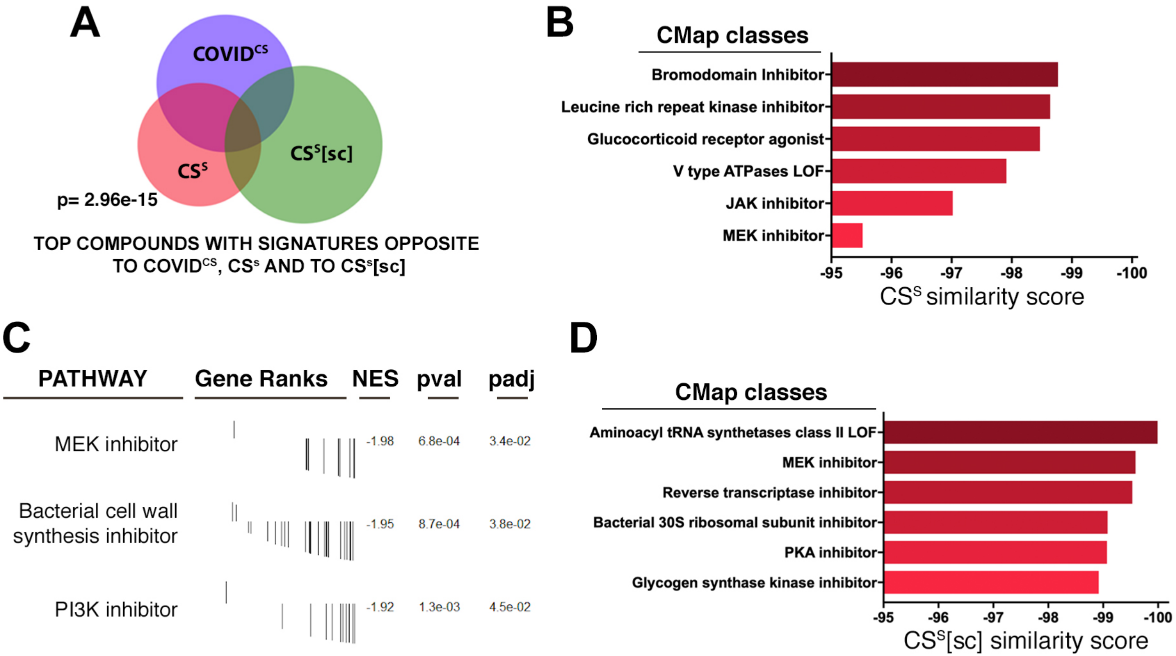

Figure S2. **Compounds triggering transcriptional signatures opposite to CS^{S} and CS^{S}[sc].** (**A**) Venn Diagram representing the overlap among the top compounds that trigger transcriptional signatures that negatively correlate to COVID^{CS}, CS^{S} and CS^{S}[sc]. The p-value was obtained with the *superexact* test. (**B**) Similarity scores of the top CMap Classes with associated transcriptional signatures that show a negative correlation to the CS^{S}. (**C**) Drug GSEA analysis of compound classes with a transcriptional signature that negatively correlates to the CS^{S}[sc]. Enriched pathways as well as their gene ranks, Normalized Enrichment Scores (NES), p-values (pval) and adjusted p-values (padj) are shown. (**C**) Similarity scores of the top CMap Classes with associated transcriptional signatures that show a negative correlation to the CS^{S}[sc].

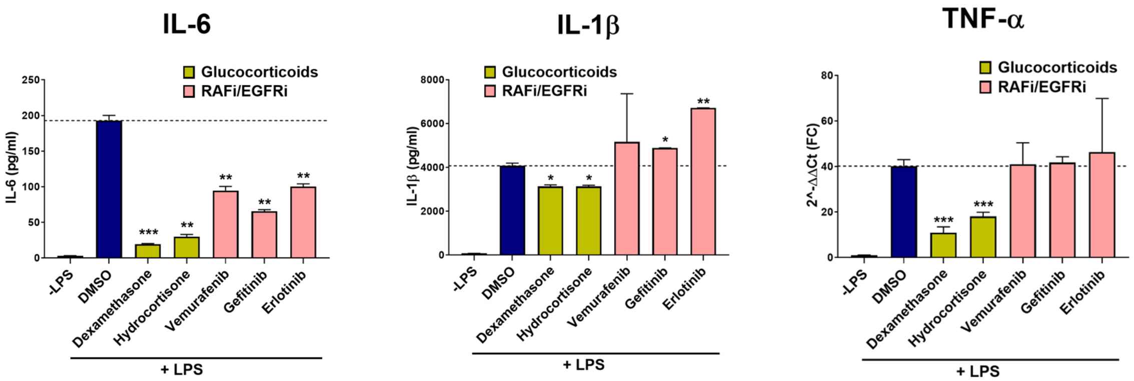

Figure S3. **Effect of RAF and EGFR inhibitors in counteracting cytokine production.** Cytokine expression in macrophages differentiated from THP1 cells upon exposure to LPS, with or without glucocorticoids (dexamethasone and hydrocortisone), a RAF inhibitor (vemurafenib) and EGFR inhibitors (gefitinib and erlotinib). IL-6 and IL-1β levels were measured by ELISA (pg/ml), and TNF-α by qPCR (2^-ΔΔCt (FC)). The experiment was repeated three times and a representative example is shown. *p<0.05, **p<0.01, ***p<0.001, ****p<0.0001 (*t* test).
